# Andrographolide attenuates imbalance of gastric vascular homeostasis induced by ethanol through glycolysis pathway

**DOI:** 10.1038/s41598-019-41417-5

**Published:** 2019-03-21

**Authors:** Huan Yao, Ziqiang Wu, Yiming Xu, Huan Xu, Guanhua Lou, Qing Jiang, Weichuan Fan, Weiming Liu, Chuan Zheng, Yongxiang Gao, Yong Wang

**Affiliations:** 1 College of Basic Medicine, Chengdu University of Traditional Chinese Medicine, Chengdu, China; 2 College Pharmacy, Chengdu University of Traditional Chinese Medicine, Chengdu, China; 3 School of Basic Medical Sciences, Guangzhou Medical University, Guangzhou, China; 4 Chengdu Tongde Pharmaceutical CO., LTD, Chengdu, China; 5 Rehabilitation research center, Beijing Boai hospital, Department of intensive care medicine, Capital Medical University Rehabilitation Academy, Beijing, China

## Abstract

Different kinds of factors contribute to gastric ulcer development which characterized by damaging gastric mucosal layer. However, gastric vascular homeostasis is not well defined and whether andrographolide has a protective function is largely unknown. The goal of this study is to investigate the potential function roles and underlying mechanism by which andrographolide regulates gastric vascular homeostasis *in vivo* and *in vitro*. Gastric ulcer animal model induced on andrographolide pretreated C57/BL6 mouse by ethanol intragastric administration. Hematoxylin and Eosin Stain, Masson’s trichrome stain and Immunohistochemistry stain performed to observe gastric vascular homeostasis, which associated hemorrhage, extracellular matrix deposition and macrophage infiltration. The activity of vascular endothelial cells were associated with the proliferation and migration, which were detected using cell counting, MTS, and wound scratch healing assay. The underlying endothelial glycolytic mechanism investigated *in vivo* and *in vitro*. Andrographolide pretreatment dramatically attenuates ethanol intragastric administration induced imbalance of gastric vascular homeostasis which characterized by severe hemorrhage, increase extracellular matrix deposition and augment macrophage infiltration. Andrographolide treatment conspicuous inhibits HUVEC-C activity characterized by suppressing proliferation and migration of endothelial cells. Mechanically, andrographolide treatment significant suppresses the expression of glycolytic genes, especially decrease PFKFB3 expression. The treatment with PFKFB3 inhibitor, 3-PO, exacerbates the inhibitory function of andrographolide on vascular endothelial cell proliferation and migration. Those data Suggests that andrographolide contributes to maintain gastric vascular homeostasis, at least partially, by inhibiting PFKFB3 mediated glycolysis pathway. Andrographolide plays a crucial role in maintaining gastric vascular homeostasis during gastric ulcer development through regulating vascular endothelial cell glycolytic pathway.

## Introduction

Gastric ulcer is open scores developed inside lining of stomach characterized by burning pain. However, the underlying mechanism for gastric ulcer development still poorly understood. One common factor contributes to the development of gastric ulcer is infected by bacterial helicobacter pylori^1^. Some other factors, such as long-term using of aspirin and certain painkillers, and habits and customs including smoking, alcohol drinking, dietary habits are contributors for gastric ulcer development^2,3^. Major causes mentioned above interrupt the balance of mucosal barriers resulting in serious inflammation^4^. However, whether the vascular system homeostasis within mucosal layer is essential for gastric ulcer development is unclear.

Andrographis paniculata is traditional used in treatment of diseases with burning symptom by its cooling specialty. Andrographis paniculata is used to treat coronary thrombotic myocardial infarction, thromboangiitis obliterans, transplantation tumor, neumonia and airway inflammation^5–7^. Andrographolide is a key principle isolated from andrographis paniculata. Andrographolide possess a strong anti-inflammatory activity through suppresses PI3K-Akt pathway and inhibition NO and PGE production^8–10^. Andrographolide inhibits smooth muscle cell proliferation via G2/M cell cycle arrest^11,12^; suppresses cell migration through Hif1α and PI3K-Akt signaling pathway^13,14^; decreases fibrosis through TGF-beta signaling pathway *et al*.^13,15^. Andrographolide inhibits gastric cancer cell proliferation^16^. However, the functional role of andrographolide on gastric ulcer development is still largely unknown.

Ethanol intragastric administration induced Gastric ulcer animal model has been widely used to explore the underlying mechanism for gastric ulcer development^17^. Ethanol intragastric administration causes mucosal damage^18,19^, as well as serious inflammatory response^20^. The gastric mucosal blood flow was interrupted in ethanol induced gastric damage^21^. The vertebrate gastric vascular wall is major composed of endothelial cells. The dysfunction of endothelial barrier leads to vascular permeability characterized serious inflammatory response^22,23^. However, whether ethanol induced gastric inflammation through impairing endothelial regulated vascular homeostasis still poorly understood.

Andrographolide has been used in treatment of metabolic syndrome^24^, such as enhancing insulin sensitivity in high-fat diet-induced obesity animal model^25,26^. Glycolytic pathway is essential for proliferation and migration of endothelial cell^27,28^. Metabolism of glucose produces ethanol, physical dosage of ethanol inhibits glucose metabolism^29^. However, whether glycolytic pathway contributes to maintain gastric vascular homeostasis in a gastric ulcer animal model induced by ethanol intragastric administration is remain unknown.

Our study indicates that andrographole treatment dramatically suppresses the expression of PFKFB3, a rate-limiting enzyme that was involved in regulating glycolysis. Taken together, those description mentioned above demonstrate that it is very likely ethanol intragastric administration initially causes imbalance of gastric mucosal vascular homeostasis, subsequently enhance macrophage infiltration associated inflammatory response, and eventually leading gastric ulcer development. Andrographolide intraperitoneal injection treatment attenuates ethanol-induced imbalance of gastric vascular homeostasis, which is, at least partially, associated with glycolysis pathway.

This study aimed to investigate the function role of andrographolide on gastric ulcer development. Ethanol intragastric administration induced gastric ulcer animal model used to explore whether andrographolide is critical to maintain gastric ulcer vascular homeostasis; Human umbilical vascular endothelial cells used to investigate whether PFKFB3 mediated glycolysis pathway in involved for andrographolide in regulating gastric ulcer development.

## Methods

The use of mice approved by the Experimental Animal Ethics Committee at Chengdu University of Traditional Chinese Medicine in accordance with NIH guidelines.

All methods used in this study carried out in accordance with manufacturer instructions or previous published papers.

### Ethanol induced gastric ulcer animal model

C57/BL6 mouse were treated with andrographolide (10 mg/kg) by intraperitoneal injection for 7 consecutive days followed by ethanol intragastric administration (100 μl/10 g). 2 hour later, the tissues of gastric was been harvested and undergoing whole mount observation or paraffin embedded.

### Cell culture HUVEC-C and HUVEC

Human umbilical vein/vascular endothelial cell line (HUVEC-C, ATCC^®^ CRL1730™) was purchased from ATCC and cultured in F-12K medium (ATCC® 30-2004™) contained 10% FBS. Human umbilical vein/vascular endothelial (HUVEC, ATCC® PCS-100-013™) was cultured in with vascular cell basal medium (ATCC, PCS-100-030) supplemented with endothelial Cell Growth Kit (ATCC, PCS-100-040).

### HUVEC cell counting

1 × 10^5^ HUVEC-C (each well) were seeded in 6-well culture plate following andrographolide treatment (5 μM/ml). Washed with PBS, trypsinized and the Cell numbers counted at 12 hours, 24 hours and 48 hours.

### HUVEC Proliferation Assay

3 × 10^3^ HUVEC-C (each well) were seeded in 96-well culture plate. The absorbance was measured using MTS proliferation assay at 490 µm.

### Scratch wound healing Assay

Treated HUVEC-C with andrographolide (5 μM/ml) for 24 hours, trypsinized and seeded into 6-well culture plate at a density of 1 × 10^6^ cells/well. A scratch across the center of the well gently and slowly made with a 10 ul pipette tip after the cell adherent. The relative gap distance monitored at different time points after crystal violet staining.

### Quantitative real time PCR analysis

Total RNA from cells was extracted using TRIzol reagent. 400 ng RNA used as template for reverse transcription with random hexamer primers using iScript cDNA synthesis kit. Real time PCR performed duplicated on ABI real time PCR system with gene specific primers listed in table (Supplementary Table S1). Relative gene expression was analysis using the 2^−∆∆ct^ method against β-actin.

### Protein preparation and Western blotting

Protein from cells were extracted using RAPI buffer containing protease inhibitor. Protein concentration was quantified using BCA assay and separated with SDS-PAGE gel. The antibody used in this study were CD31 (Biocare; mouse, 1:200); Mac-2 (Antidodies; rabbit, 1:200); PCNA (Cell Signaling Technology; mouse, 1:1000); β-actin (Cell Signaling Technology; mouse, 1:3000); AKT (Cell Signaling Technology; rabbit, 1:2000); pAKT (Cell Signaling Technology; rabbit, 1:2000); PFKFB3 (Proteintech; rabbit, 1:1000). Images captured by using ImageQuan LAS4000 Imaging Station (GE) and the densities of bands were quantified using the ImageQuant TL software (GE).

### Hematoxylin and Eosin (HE) Stain and Immunohistochemistry (IHC)

Gastric tissues fixed with 4% paraformaldehyde overnight at 4 °C and paraffin embedded, 5-μm thickness of slides collected and deparaffinized. Hematoxylin/eosin (HE) staining performed as previously described^30^. For IHC staining, the deparaffinized slides were treated with citric acid and antigenic unmasked at 98 °C for 5–10 minutes, incubated with primary antibodies overnight at 4 °C, followed by incubation with biotinylated secondary antibody at room temperature for 1 hour (Vector Laboratories, 1:200), and ABC solution (Vector Laboratories, Burlingame, CA) for 30 minutes at room temperature. Expression of the targets visualized after DAB solution added.

### Statistics

Quantitative data were expressed as mean ± SEM. Comparisons between 2 groups were analysis by unpaired student’s t test using prism software. A value of P < 0.05 was considered statistically significant.

## Results

### Andrographolide attenuates ethanol-induced imbalance of gastric vascular homeostasis

**Figure 1:**
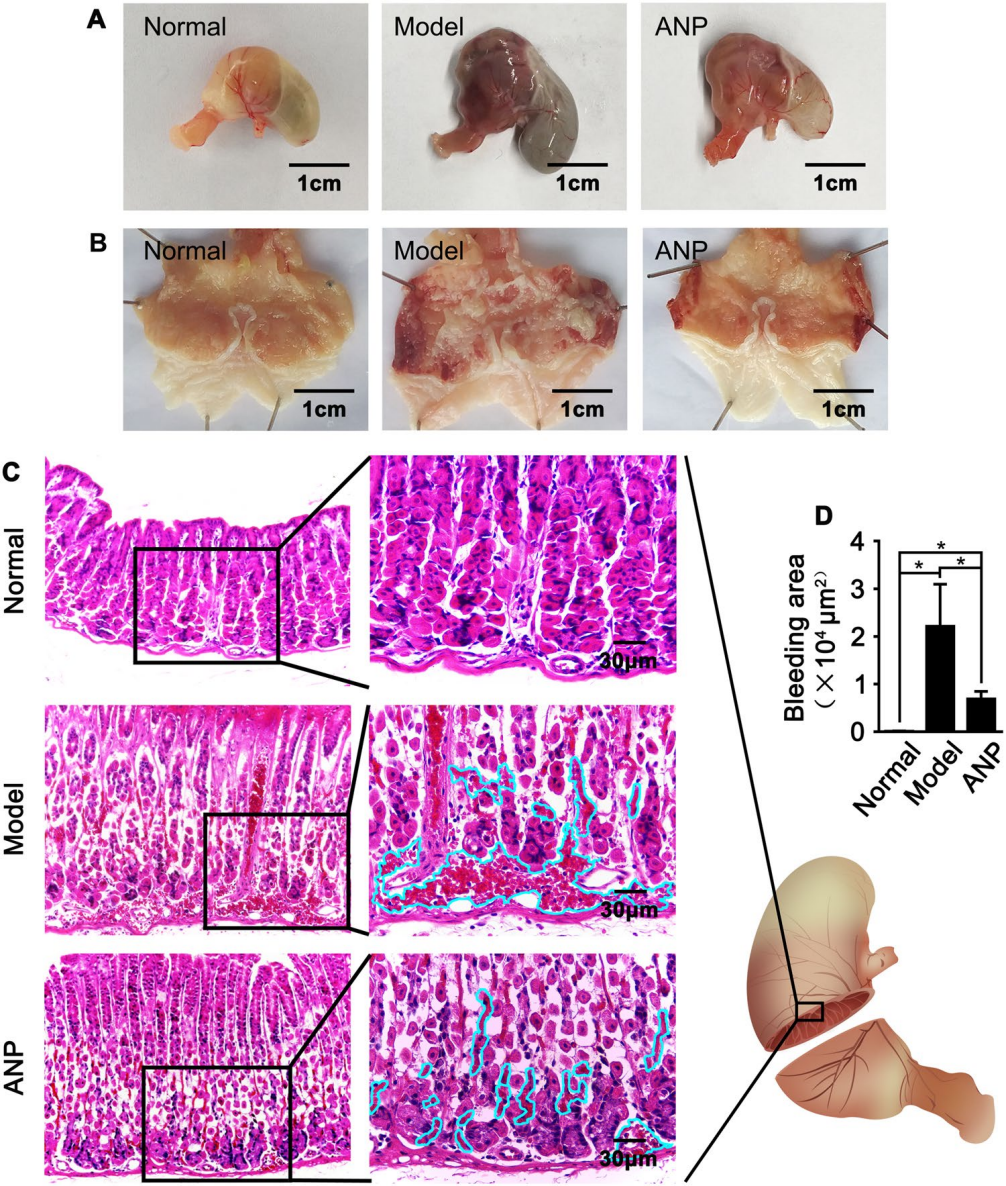
Andrographolide attenuates ethanol-induced imbalance of gastric vascular homeostasis. (**A**) Representative whole mount images of gastric ulcer induced by ethanol intragastric administration after andrographolide pretreatment and the cut-opened images were displayed in (**B**). (**C**) HE staining of the gastric revealed hemorrhage occurred in mucosa layer. (**D**) The area of hemorrhage in gastric mucosa layer were quantitative by Image J. Data were presented as mean ± SEM (n = 10, *P < 0.05). (Schematic drawing in (**C**) was draw by Huan Yao).

To determine the role of Andrographolide in gastric ulcer vascular homeostasis regulation, we pretreated C57/BL6 mice with andrographolide for 7 consecutive days following ethanol intragastric administration to induce gastric ulcer model. Ethanol suppresses gastric emptying resulting in gastric enlargement. Surface bulges observed in the mucosal surface characterized by edema and serious hemorrhage. Pretreatment with Andrographolide significant suppresses hemorrhage as well as edema (Fig. 1A,B). To verify the hemorrhage symptom, we performed Hematoxylin and Eosin staining on gastric paraffin embedded slides. Our data exhibits the majority of hemorrhage occurred in submucosa layer. However, andrographolide treatment dramatically inhibits ethanol induced submucosa hemorrhage (Fig. 1C,D).

### Andrographolide attenuates gastric mucosa extracellular matrix deposition

**Figure 2:**
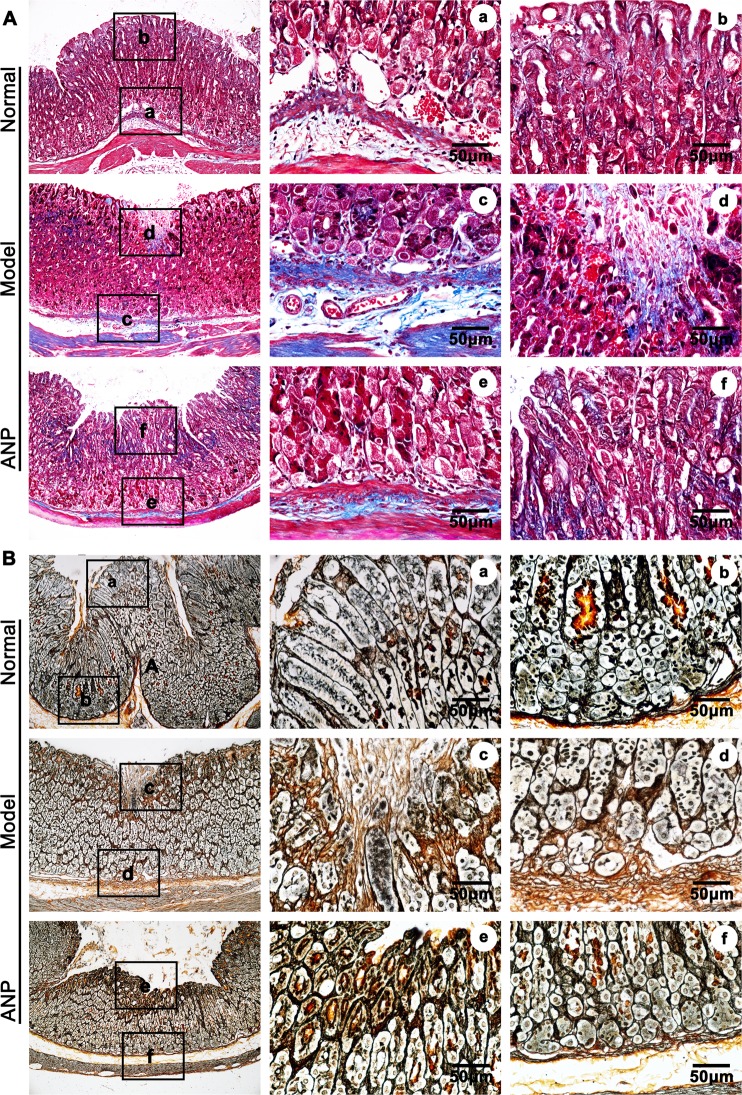
Andrographolde decreases expression of extracellular matrix deposition. (**A**) Representative image of collagen within mucosa layer using Masson’s trichrome staining. (**B**) Representative image of collagen and reticulin fiber within mucosa layer using Gomori methenamine silver stain. (Normal group means without treatment, Model group refer to vehicle retreatment and following ethanol intragastric administration, and ANP group refer to ANP pretreatment and following ethanol intragastric administration).

To determine whether the hemorrhage occurrence was due to imbalance of vascular homeostasis, we sought to determine expression of extracellular matrix genes using quantitative real time PCR in andrographolide pretreated HUVEC-C. We first performed WST-1 assay to optimize the dose of andrographolide for *in vitro* study (Supplementary Fig. S1). After andrographolide treatment, extracellular matrix related genes, including versican0, has3, as well as fibronectin, collagen I were significant decreased compared to that of vehicle treatment (Supplementary Fig. S2A,B). Our masson’s trichrome staining and Gomori methenamine silver stain data demonstrates that andrographolide treatment down-regulates collagen deposition in gastric ulcer animal model (Fig. 2A,B). Those results indicating that andrographolide play an important role in regulating extracellular matrix deposition.

### Andrographolide impairs endothelial cells activation

**Figure 3:**
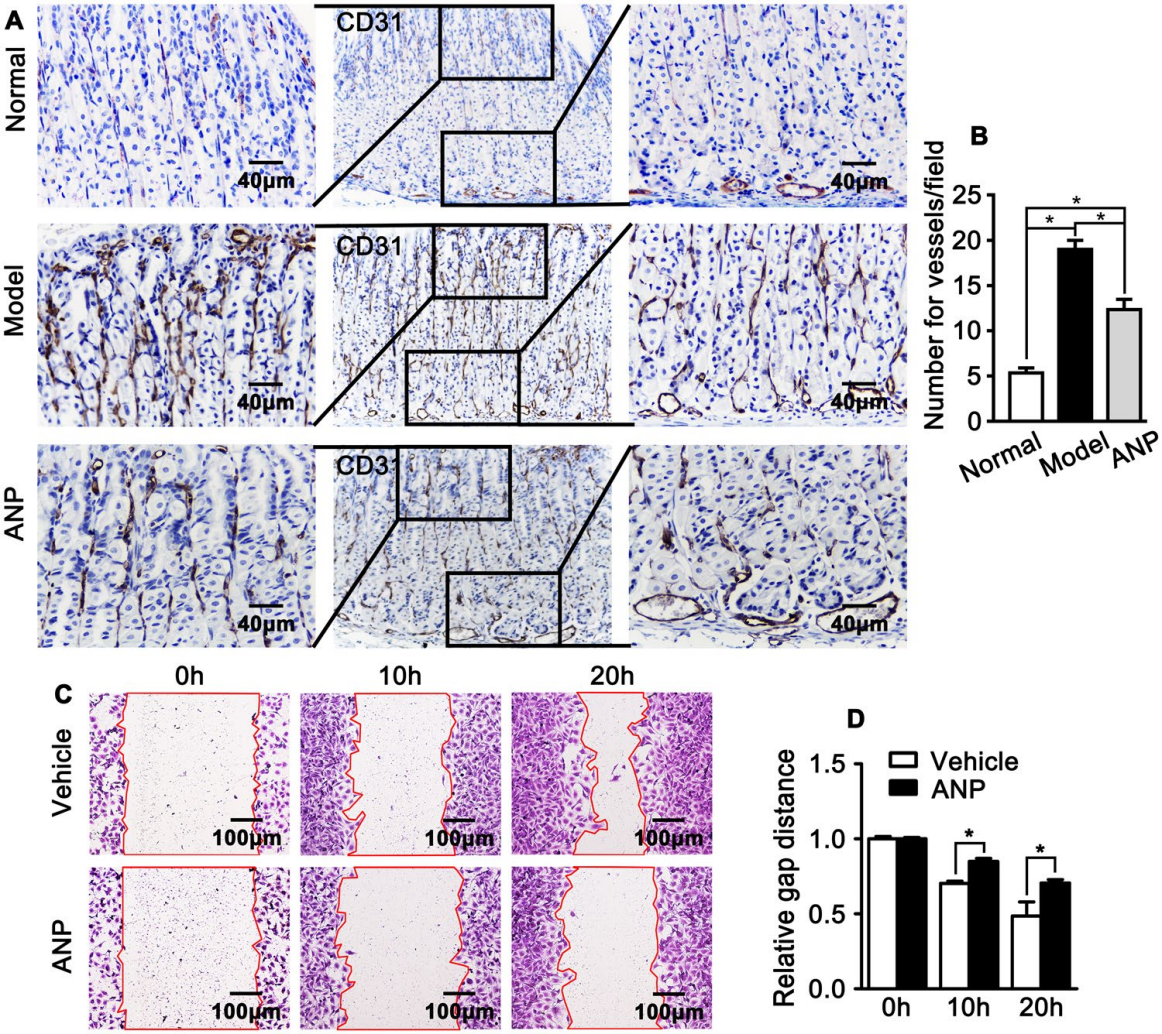
Andrographolide suppresses endothelial activity. (**A**) Representative Immunohistochemistry staining of endothelial specific gene CD31 in gastric wall and quantification of the capillary numbers in mucosa layer displayed in (**B**). Data were presented as mean ± SEM (n = 8, *P < 0.05). (**C**) Scratch wound healing assay was performed to observe the migration of HUVEC-C treated with andrographolide at different time point visualization by crystal violet staining and relative gap distance was quantified in (**D**). Data presented as mean ± SEM, each group repeated triplicated.

Endothelial cell is one of the key component for gastric vascular system. Endothelial cell activation plays pivotal role in maintaining vascular homeostasis. Whether andrographolide is essential for maintain gastric vascular homeostasis still unclear. We performed IHC stain against endothelial specific gene CD31 to observe endothelial cell activation. Ethanol markedly promotes endothelial cell activation characterized by enhanced CD31 expression and increased capillary numbers in both mucosal and submucosal layer. However, andrographolide treatment significant suppresses CD31 expression and capillary numbers (Fig. 3A,B).

### Andrographolide treatment suppresses HUVEC migration

Migration of endothelial cell is essential for angiogenesis, tumorigenesis and vascular permeability^31^. We next sought to investigate whether andrographolide plays an essential role in regulating endothelial cell migration. Andrographolide treatment inhibits endothelial cell migration characterized by overt scratch gap existing at 20 hours after scratch damage in a scratch wound healing migration assay (Fig. 3C,D).

### Andrographolide suppresses HUVEC proliferation

**Figure 4:**
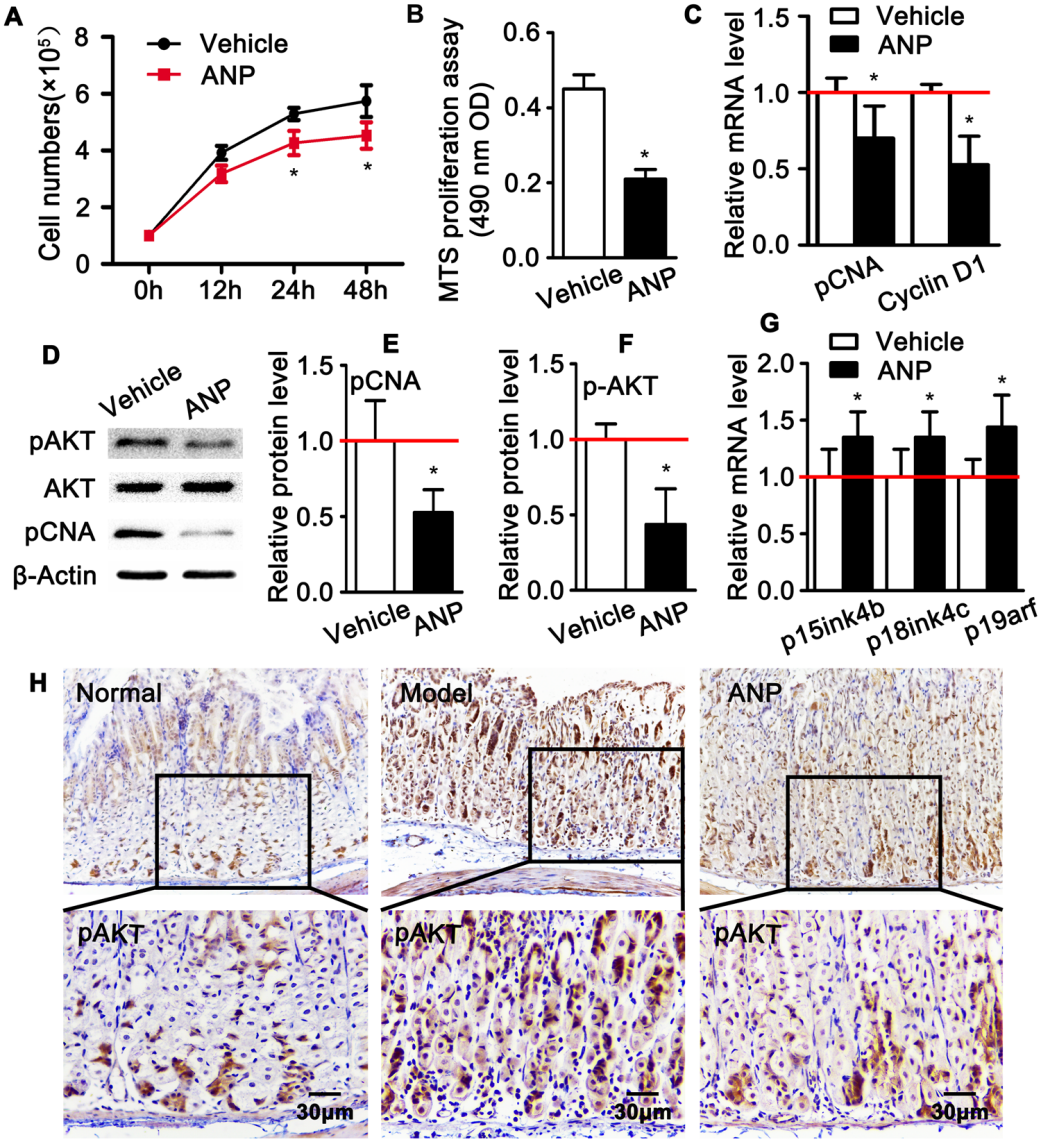
Andrographolide suppresses endothelial cells proliferation. (**A**) The growth of HUVEC-C treated with andrographolide was accomplished by cell counting. (**B**) MTS proliferation assay was performed to observe the HUVEC-C viability after treated with andrographolide at a concentration of 5 μM for 24 hours. (**C**) Quantitative real time RT-PCR analysis of the expression of PCNA and Cyclin D1 in HUVEC-C treated with andrographolide. (**D**) Western blot was performed to observe the proliferation activity of HUVEC-C treated with andrographolide and quantification data were displayed in (**E**,**F**). (**G**) Quantitative real time RT-PCR analysis the expression of cell cycle negative regulated gene. All data presented as mean ± SEM, *P < 0.05 was considered significant. The experiments repeated three times. (**H**) Representative IHC images stained against anti-phosphorylated AKT.

Previously data demonstrate that andrographolide inhibits cells proliferation, including smooth muscle cells and cerebral endothelial cells. Therefore, we treated HUVEC-C with andrographolide and performed cell number counting experiment. Andrographolide treatment inhibits endothelial cell growth exhibiting dramatically decreased the cell numbers compared to the vehicle treatment (Fig. 4A). Andrographolide overt inhibits HUVEC-C viability based on a MTS proliferation assay (Fig. 4B). Quantitative real time PCR and Western blot were used to observe proliferative marker genes, such as PCNA and phosphorylated AKT. Our data indicates that andrographolide suppresses proliferation of vascular endothelial cell (Fig. 4C–F). The *in vivo* study confirms that andrographolide dramatically suppresses expression of phosphorylated AKT within mucosa layer (Fig. 4H). Furthermore, andrographolide treatment leads to increase expression of cell cycle negative regulator, such as P15ink4b, P18ink4c and P19arf (Fig. 4G). Taken together, data mentioned above indicates that andrographolide inhibits HUVEC-C proliferation, partially through induction cell cycle negative regulated genes.

### PFKFB3 mediated glycolysis is essential for andrographolide to maintain gastric vascular homeostasis

**Figure 5:**
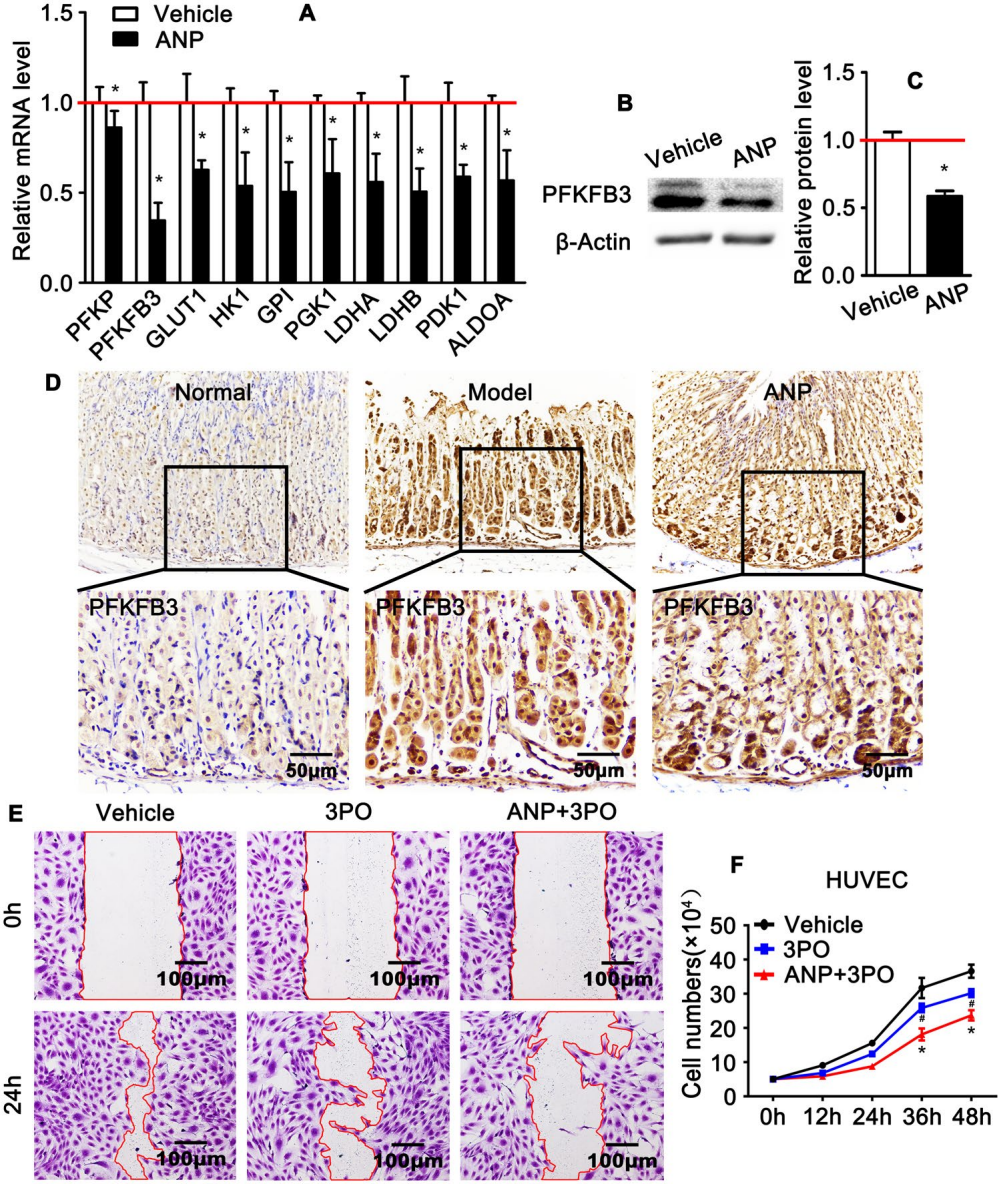
Andrographolide suppresses endothelial PFKFB3 mediated glycolysis pathway. (**A**) Quantitative real time RT-PCR analysis of the expression of glycolysis related genes in HUVEC-C following andrographolide treatment. (**B**) Western blot analysis PFKFB3 expression in HUVEC-C after andrographolide treatment and the quantification data displayed in (**C**). All data presented as mean ± SEM, *P < 0.05 was considered significant. The experiments repeated three times. (**D**) IHC was performed to observe the expression of PFKFB3 in gastric wall. (**E**,**F**) HUVEC treated with 3-PO, a PFKFB3 inhibitor, would scratch and cell number accounting experiment performed to detect the proliferation and migration.

Our previously data demonstrate that glycolysis pathway is essential for endothelial cell migration and proliferation^28^. However, whether glycolysis pathway is essential to maintain gastric vascular homeostasis during gastric ulcer development still unclear. We further sought to explore whether andrographolide plays a critical role in regulating endothelial glycolysis in an ethanol-induced gastric ulcer animal model. Quantitative real time PCR performed to observe the expression of glycolysis regulated genes, including PFKP, PFKFB3, GLUT1, HK1, GPI, PGK1, LDHA, LDHB, PDK1 and ALDOA. Andrographolide suppresses those glycolysis regulators expression compare to that of vehicle treatment, especially PFKFB3, a rate-limiting enzyme contributing to mediating glycolytic pathway (Fig. 5A). The expression of PFKFB3 protein level is also downregulated following andrographolide treatment (Fig. 5B,C). Similarly, PFKFB3 expression further confirmed using IHC staining on gastric slide from ethanol induced gastric ulcer model (Fig. 5D). Our data demonstrate that andrographolide maintains gastric vascular homeostasis possibly through PFKFB3 mediated glycolysis pathway. In order to detect this hypothesis, we treated HUVEC with 3-PO, a PFKFB3 inhibitor, and performed the wound healing and cell accounting experiment again. Our data suggests that 3-PO treatment exacerbate the inhibitory function of andrographolide on vascular endothelial cell proliferation and migration (Fig. 5E,F). Those results mentioned above demonstrate that andrographolide maintains mucosa layer vascular hemostasis through PFKFB3 mediated glycolytic pathway.

### Andrographolide attenuates gastric submucosa macrophage infiltration

**Figure 6:**
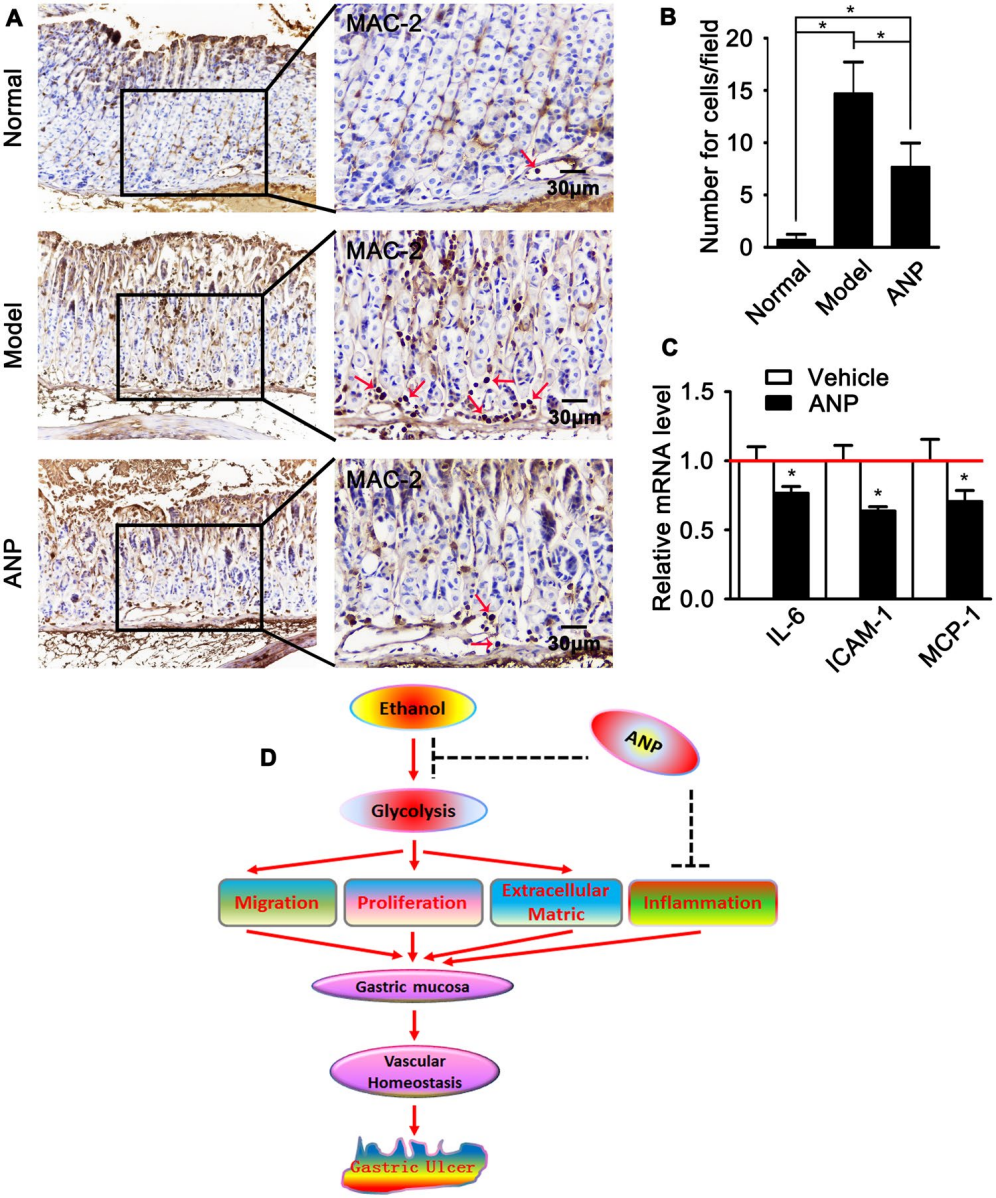
Andrographolide inhibits gastric inflammation. (**A**) Representative Immunohistochemistry staining against Mac-2 on sections of gastric ulcer from mice pretreated with vehicle or andrographolide. The number of Mac-2 quantified in (**B**). Data were presented as mean ± SEM (n = 10, *P < 0.05). (**C**) Quantitative real time RT-PCR analysis of inflammatory response genes in HUVEC-C pretreated with andrographolide (5 μM/ml) for 24 hours. Data presented as mean ± SEM. Experiments repeated twice. *P < 0.05. (**D**) Schematic diagram demonstrates the function role of andrographolide in attenuating imbalance of vascular homeostasis in a gastric ulcer animal model induced by ethanol intragastric administration through endothelial glycolytic pathway.

Emerging evident demonstrate that inflammatory plays a crucial role during gastric ulcer development. However, the underling mechanism causing inflammation in gastric mucosa is not clearly. We determined macrophage infiltration in gastric mucosa by IHC staining against Mac2 antibody. Ethanol promotes macrophage infiltration in both layers of gastric mucosa and submucosa. However, macrophage infiltration observably inhibited by andrographolide pretreatment (Fig. 6A,B). The expression of inflammatory genes, including IL-6, Icam1, MCP-1, were also suppressed in HUVEC-C following andrographolide treatment analyzed by quantitative real time PCR (Fig. 6C).

Taken together, data described above demonstrate that andrographolide attenuates ethanol induced endothelial glycolysis resulting in maintain gastric vascular homeostasis characterized by decreased endothelial migration, proliferation and extracellular matrix deposition, consequently suppresses macrophage infiltration, which eventually induction of gastric ulcer development (Fig. 6D).

## Discussion

This study for the first time provides evidence that demonstrates a critical protective role of andrographolide in gastric ulcer development via maintaining gastric vascular homeostatis. A number of factors contributes to gastric ulcer development have been defined, such as bacterial helicobacter pylori infection, drug abuse, endocrine dyscrasia, heavy drinking and so on, which impairs mucosal barrier through disturbing gastric acid secretion characterized by hemorrhage and serious inflammatory response^32,33^. However, we observed severe hemorrhage within submucosal layer with intact mucosal layer, as well as abundant macrophage infiltration (Fig. 6A). Those data suggests, except damage of mucosal layer, inflammatory cells infiltration from circulation due to vascular homeostasis impairment also contribute to gastric ulcer development.

Imbalance of vascular homeostasis results in blood leakage and inflammatory response. We further sought to investigate whether andrographolide is essential for gastric vascular homeostasis regulation. Andrographolide pretreatment significant decreases extracellular matrix deposition (Fig. 2A,B). However, further study still need to explore the underlying mechanism. Andrographolide treatment prominent inhibits endothelial cells activity characterized by suppression expression of endothelial specific marker gene CD31 in mucosal layer of gastric ulcer animal model. Andrographolide treatment conspicuously inhibiting endothelial cell’s proliferation, as well as migration (Figs 3C and 4). This function is partially through regulating cell cycle negative regulators. Ethanol treatment leads to vascular unstabilization due to increased endothelial activity, as well disorganization of extracellular matrix deposition. Which make sense in principle that the hemorrhage and macrophage infiltration exhibited within submucosal layer.

In this study we identified a novel mechanism whereby andrographolide attenuates imbalance of gastric vascular homeostasis induced by ethanol intragastric administrate through suppressing PFKFB3 mediated glycolytic pathway. Our previously data demonstrate endothelial cell utilizes aerobic glycolysis to generate energy to maintain cellular functions^28^. Whether glycolytic pathway is crucial for andrographolide regulation gastric vascular homeostasis is never been investigated. Andrographolide treatment prominent suppresses glycolytic genes expression *in vivo* and *in vitro* (Fig. 5). However, the ubiquitin expression of glycolytic enzyme PFKFB3 in gastric mucosal layer suggests that beyond maintain vascular homeostasis, glycolysis pathway is likely contributes to inhibiting gastric acid secretion, repairing damaged mucosal layer.

PFKFB3 plays a pivotal role in the regulation of glycolytic flux. Enhanced glycolysis been reported in several proliferative cell types, transformed cell types, tumor cells, adipocytes and endothelial cells. Multiples molecules contribute to regulate the intracellular glycolytic flux. PFKFBs catalyze the synthesis and degradation of fructose 2, 6-bishosphate (F2, 6 BP), a powerful activator of 6-phosphofructo-1-kinase, the rate-limiting enzyme in glycolytic flux. Four isoforms of PFK-2 been identified in mammal cells, which encoded by the genes PFKFB1, PFKFB2, PFKFB3 and PFKFB4. With high kinase-to-phosphatase activity ratio, PFK2 that encoded by PFKFB3 plays a key role in the regulation of glycolytic flux. PFKFB3 mediated glycolytic flux in endothelial cells is critical in regulation endothelial cell proliferation, migration and endothelial tip cell sprouting^27,28^.

Interesting, our *in vitro* study demonstrate that treatment of andrographolide suppresses PFKFB3 expression, as well as macrophage infiltration within mucosa layers (Supplementary Fig. S3). We sought to observe whether glycolytic pathway is critical to maintain mucosa layer homeostasis through inflammatory reaction dependent or independent pathway, we detected the expression of PFKFB3 and macrophage infiltration at different time point after ethanol intragastric administration. Our data shown demonstrate that the expression of PFKFB3 in vascular endothelial within mucosa layer is increased at 30 minute, and high expression level is sustained to 2 hour. However, no macrophage infiltration detected at 30 minute. At the later stage, macrophage infiltration observed within mucosa layer (Supplementary Fig. S4). The results indicates that andrographolide attenuates ethanol induced endothelial glycolysis resulting in maintain gastric vascular homeostasis characterized by decreased endothelial migration, proliferation and extracellular matrix deposition, consequently suppresses macrophage infiltration, which eventually induction of gastric ulcer development.

In summary, this study demonstrates a protective function of andrographolide during gastric ulcer development, and identifies a novel mechanism via endothelial glycolysis pathway regulated by PFKFB3 to maintain gastric vascular homeostasis.

## Supplementary information

Dataset 1
