# Supplementary material for: Andrographolide attenuates imbalance of gastric vascular homeostasis induced by ethanol through glycolysis pathway
Source: Sci Rep. 2019 Mar 21;9:4968. doi: 10.1038/s41598-019-41417-5 (PMC6428857; doi:10.1038/s41598-019-41417-5)
Supplement: Supplementary file 1 — Dataset 1 [file 41598_2019_41417_MOESM1_ESM.docx]

**Supplementary information**

**Andrographolide attenuates imbalance of gastric vascular homeostasis induced by ethanol through glycolysis pathway**

Huan Yao^1*^, Ziqiang Wu^2*^, Yiming Xu^3^, Huan Xu^1^, Guanhua Lou^2^, Qing Jiang^2^, Weichuan Fan^4^, Weiming Liu^5^, Chuan Zheng^1^, Yongxiang Gao^1#^, Yong Wang^1#^

^1^ College of Basic Medicine, Chengdu University of Traditional Chinese Medicine, Chengdu, China. ^2^ College Pharmacy, Chengdu University of Traditional Chinese Medicine, Chengdu, China. ^3^ School of Basic Medical Sciences, Guangzhou Medical University, Guangzhou, China. ^4^ Chengdu Tongde Pharmaceutical CO., LTD, Chengdu, China. ^5^ Rehabilitation research center, Beijing Boai hospital, Department of intensive care medicine, Capital Medical University Rehabilitation Academy, Beijing, China.

* Equally contributed to this study

^#^Address for Correspondence:

Yong Wang, PhD

Basic Medical College, Cheng Du University of Traditional Chinese Medicine, Chengdu, Sichuan China

Email: yongwang1008@hotmail.com

Yongxiang Gao, PhD

Basic Medical College, Cheng Du University of Traditional Chinese Medicine, Chengdu, Sichuan China

Email: yxgaocd@163.com

**Supplementary Methods**

**Ethanol induced gastric ulcer animal model**

The use of mice approved by the Experimental Animal Ethics Committee at Chengdu University of Traditional Chinese Medicine in accordance with NIH guidelines.

Andrographolide dissolved in DMSO to a concentration of 0.1g/ml and stored in -20℃. For the intragastric administration, the dissolved andrographolide further diluted with intralipid (a 20% I.N. fat emulsion).

Gastric ulcer model were generated as previously described in details[^1^](#_ENREF_1)^,^[^2^](#_ENREF_2). 8 to 10 weeks old C57/BL6 mouse were treated with andrographolide (10mg/kg) by intraperitoneal injection for 7 consecutive days, fasted overnight, followed by absolute ethanol intragastric administration (100μl/10g). 2 hour later, the tissues of gastric was been harvested and undergoing whole mount observation or paraffin embedded.

**Cell culture HUVEC-C**

Human umbilical vein/vascular endothelial cell line (HUVEC-C, ATCC^®^ CRL­1730™) was purchased from ATCC and cultured in F-12K medium (ATCC^®^ 30-2004™) contained 10% FBS. Human umbilical vein/vascular endothelial (HUVEC, ATCC® PCS-100-013™) was cultured in with vascular cell basal medium (ATCC, PCS-100-030) supplemented with endothelial Cell Growth Kit (ATCC, PCS-100-040).

**HUVEC cell counting**

Cell counting were performed as described in our previous report[^3^](#_ENREF_3). 1×10^5^ HUVEC-C (each well) were seeded in 6-well culture plate following andrographolide treatment (5μM/ml) or vehicle. Washed with PBS, trypsinized and the Cell numbers counted at 12 hours, 24 hours and 48 hours. For each time point, triplicate for each group.

**HUVEC Proliferation Assay**

3×10^3^ HUVEC-C (each well) were seeded in 96-well culture plate. Treated the cells with andrographolide (5μM/ml) or vehicle overnight. Added MTS reagent and incubated for 0.5 to 4 hours at 37°C in standard culture conditions. The absorbance was measured using MTS proliferation assay at 490 nm.

**Scratch wound healing Assay**

Western blot were performed as described in our previous report[^4^](#_ENREF_4). HUVEC-C treated with andrographolide (5μM/ml) or vehicle for 24 hours, trypsinized and seeded into 6-well culture plate at a density of 1×10^6^ cells/well. A scratch across the center of the well gently and slowly made with a 10ul pipette tip after the cell adherent. The relative gap distance monitored at different time points after crystal violet staining.

**Quantitative real time PCR analysis**

Quantitative real time PCR performed as described in our previous report[^5^](#_ENREF_5). Total RNA from cells was extracted using TRIzol reagent. 400ng RNA were using as template for reverse transcription with random hexamer primers using iScript cDNA synthesis kit. Real time PCR performed duplicated on ABI real time PCR system with gene specific primers listed in table (table 1). Relative gene expression was analysis using the 2^-∆∆ct^ method against β-actin.

**Protein preparation and Western blotting**

Western blot performed as described in our previous report[^6^](#_ENREF_6). Protein from cells were extracted using RAPI buffer containing protease inhibitor. Protein concentration was quantified using BCA assay and separated with SDS-PAGE gel. The antibody used in this study were CD31 (Biocare; mouse, 1:200); Mac-2 (Biocompare; rabbit, 1:200); PCNA (Cell Signaling Technology; mouse, 1:1000); β-actin (Cell Signaling Technology; mouse, 1:3000); AKT (Cell Signaling Technology; rabbit, 1:2000); pAKT (Cell Signaling Technology; rabbit, 1:2000); PFKFB3 (Proteintech; rabbit, 1:1000). Images captured by using ImageQuan LAS4000 Imaging Station (GE) and the densities of bands were quantified using the ImageQuant TL software (GE).

**Hematoxylin and Eosin (HE) Stain and Immunohistochemistry (IHC)**

HE and IHC staining performed as described in our previous report[^7^](#_ENREF_7). Gastric tissues fixed with 4% paraformaldehyde overnight at 4°C and paraffin embedded, 5-um thickness of slides collected and deparaffinized. Hematoxylin/eosin (HE) staining performed as previously described. For IHC staining, the deparaffinized slides were treated with citric acid and antigenic unmasked at 98°C for 5-10 minutes, incubated with primary antibodies overnight at 4°C, followed by incubation with biotinylated secondary antibody at room temperature for 1 hour (Vector Laboratories, 1:200), and ABC solution (Vector Laboratories, Burlingame, CA) for 30 minutes at room temperature. Expression of the targets visualized after DAB solution added.

**Statistical analysis**

Quantitative data were expressed as mean±SEM. Comparisons between 2 groups were analysis by unpaired student’s t test. Comparisons among 3 groups were performed by one-way ANOVA. A value of P<0.05 was considered statistically significant.

**Reference**

1 Li, W. *et al.* Protective effect of tetrahydrocoptisine against ethanol-induced gastric ulcer in mice. *Toxicology and applied pharmacology* **272**, 21-29, doi:10.1016/j.taap.2013.05.035 (2013).

2 Oates, P. J. & Hakkinen, J. P. Studies on the mechanism of ethanol-induced gastric damage in rats. *Gastroenterology* **94**, 10-21 (1988).

3 Liu Z *et al.* Endothelial adenosine a2a receptor-mediated glycolysis is essential for pathological retinal angiogenesis. *Nat Commun.* **8**, 584 (2017).

4 Xiaobo Wang *et al.* Induction of yes-associated protein expression after arterial injury is crucial for smooth muscle phenotypic modulation and neointima formation. *Arterioscler Thromb Vasc Biol. 2012 Nov;32(11):2662-9.* **32**, 2662-2669 (2012).

5 Xu, Y. *et al.* Intracellular adenosine regulates epigenetic programming in endothelial cells to promote angiogenesis. *EMBO molecular medicine* **9**, 1263-1278, doi:10.15252/emmm.201607066 (2017).

6 Xu Y *et al.* Regulation of endothelial intracellular adenosine via adenosine kinase epigenetically modulates vascular inflammation. *Nat Commun.* **8**, 943 (2017).

7 Wang, Y. *et al.* Deletion of yes-associated protein (YAP) specifically in cardiac and vascular smooth muscle cells reveals a crucial role for YAP in mouse cardiovascular development. *Circulation research* **114**, 957-965, doi:10.1161/CIRCRESAHA.114.303411 (2014).

**Supplementary figure legends**

**Supplementaty Table 1**. List of primer sequences used in the study for real time PCR. HUVEC-C treated with andrographolide (5μM/ml) or vehicle for 24 to 48 hours, total RNA was extracted using TRIzol reagent, and the following primers used to observe the transcription level.

**Supplementary Figure 1**. Optimal dose of Andrographolide selected for in vitro study. Cells treated with ANP for 36 hours and cell viability quantified by WST1 assay at 450nm.

**Supplementary Figure 2**. Andrographolide decreases expression of extracellular matrix related genes. (A). Quantitative real time RT-PCR analysis of extracellular matrix related genes in HUVEC-C pretreated with andrographolide (5 μM/ml) for 36 hours. (B). Quantitative real time RT-PCR analysis of extracellular matrix related genes in HUVEC pretreated with andrographolide (5 μM/ml) for 36 hours. Data presented as mean ± SEM. Experiments repeated twice. * P < 0.05.

**Supplementary Figure 3**. Andrographolide suppresses endothelial glycolysis pathway and inflammation. Representative Immunohistochemistry staining against MAC-2 or PFKFBE on sections of gastric ulcer from mice pretreated with vehicle or andrographolide.

**Supplementary Figure 4**. Ethanol activates endothelial glycolysis and inflammation and causes hemorrhage at different times. (A). Representative images from immunohistochemistry staining against MAC-2, PFKFB3 and H&E staining of normal gastric mucosa layer. (B). Representative images from immunohistochemistry staining against MAC-2, PFKFB3 and H&E staining of gastric mucosal layer after ethanol intragastric administration for 30 minutes and 2 hours.
